# Functional and Biological Role of Endothelial Precursor Cells in Tumour Progression: A New Potential Therapeutic Target in Haematological Malignancies

**DOI:** 10.1155/2016/7954580

**Published:** 2015-12-14

**Authors:** Antonia Reale, Assunta Melaccio, Aurelia Lamanuzzi, Ilaria Saltarella, Franco Dammacco, Angelo Vacca, Roberto Ria

**Affiliations:** Department of Biomedical Sciences and Human Oncology, Section of Internal Medicine and Clinical Oncology, University of Bari Medical School, 70124 Bari, Italy

## Abstract

It was believed that vasculogenesis occurred only during embryo life and that postnatal formation of vessels arose from angiogenesis. Recent findings demonstrate the existence of Endothelial Precursor Cells (EPCs), which take partin postnatal vasculogenesis. EPCs are recruited from the bone marrow under the stimulation of growth factors and cytokines and reach the sites of neovascularization in both physiological and pathological conditions such as malignancies where they contribute to the “angiogenic switch” and tumor progression. An implementation of circulating EPCs in the bloodstream of patients with haematological malignancies has been demonstrated. This increase is strictly related to the bone marrow microvessel density and correlated with a poor prognosis. The EPCs characterization is a very complex process and still under investigation. This literature review aims to provide an overview of the functional and biological role of EPCs in haematological malignancies and to investigate their potential as a new cancer therapeutic target.

## 1. Introduction

Blood vessels arise through two different mechanisms: vasculogenesis and angiogenesis. Vasculogenesis is the process of the* de novo *formation of blood vessels, which usually occurs during embryonic life but it can also happen in postnatal life in particular conditions; angiogenesis instead is the process of vessels formation from preexisting ones [1].

The most important cells which take part in prenatal vasculogenesis are haemangioblasts, which derive from mesenchymal stem cells, and represent the precursors of endothelial and haematopoietic cells.

During embryogenesis, between days 21 and 32, when the yolk sac develops, haemangioblasts form aggregates, also known as bloods islands. In this early process, haemangioblasts form tight junction through each other, creating a primary tube formation with a lumen; then perivascular cells resembling pericytes pass into the early lumen, and finally in the late stage of vasculogenesis the development of a basal lamina induces the separation of the vessel lumen delimited by endothelial cells from perivascular cells [2].

Before the first identification of EPCs by Asahara et al. [3] in 1997, it was believed that vasculogenesis occurred only during embryo life. Since the identification of circulating EPCs, the idea of postnatal vasculogenesis was enhanced and increasing evidence suggests the contribution of EPCs in several diseases such as myocardial ischemia, stroke, retinopathy, and peripheral vascular disease and in tumor growth and progression. Considering the importance of EPCs and their involvement in postnatal vasculogenesis in both physiological and pathological conditions, many authors have investigated the role and the characterization of EPCs in order to better understand their specific markers and the mechanisms regulating their function and differentiation [4].

During postnatal vasculogenesis, EPCs are recruited from the BM, into the blood circulation to the site of damage under the stimulation of hypoxia, growth factors (GFs), and cytokines and here they differentiate into mature endothelial cells (ECs). Evidences show, that after myocardial infarction or ischemia, neovascularization occurs and promotes regeneration of cardiac and vasculature structures; thus clinical studies have been conducted in order to infuse autologous EPCs with the aim to augment the number of EPCs, favoring the remodeling process which physiologically occurs after a cardiac damage. Many studies have demonstrated the ability of EPCs to transdifferentiate into cardiomyocytes, contributing actively to the regeneration of the tissue [5].

The involvement of EPCs in wound healing is well known. Neovascularization is essential for the survival and remodeling of injured tissue. After wound healing, the secretion of both inflammatory cytokines and growth factors recruits immune cells and also EPCs, which proliferate and induce the formation of the new blood vessel and tissue regeneration which finally is remodeled [6].

Many studies also show the importance of EPCs in bone formation and osteogenesis: deficient vasculogenesis seems to be responsible for the fail of bone restoration. As well as in postmyocardial infarction, EPCs CD34+ cells are also able to transdifferentiate in osteoblasts taking part in osteogenesis themselves [7].

Finally vasculogenesis takes part also in pathological conditions such as tumor growth and is involved in tumor progression. A typical phase of both solid and haematological malignances is the so-called “angiogenic switch” which is the transition from the avascular phase to the vascular and more aggressive phase of tumor growth [8]. Vasculogenesis and angiogenesis represent two critical mechanisms which ensure tumor progression providing nutrients, growth factors, and oxygen to tumor site, favoring also metastatic processes [9]. Thus high microvessel density (MVD) correlates with poor prognosis and a reduced survival expectation [10].

## 2. EPCs Characterization and Biology

EPCs were first identified and isolated by Asahara et al. [3] in 1997. They isolated CD34 and vascular growth factor receptor 2 (VEGFR2) positive haematopoietic progenitor cells from adults that could differentiate into cells with an endothelial phenotype and take part in neovascularization.

Moreover EPCs have been identified as bone marrow- (BM-) derived EPCs [11] and they take part in neovascularization in different tissue injuries and remodeling.

The characterization, the identification, and the origin of EPCs are now more defined.

EPCs are part of a big heterogeneous group of cells that share the same precursor within BM and are able to induce endothelial differentiation in peripheral blood. Thus the word “EPCs” can be used to recognize cells belonging to several steps ranging from primitive haemangioblasts to completely differentiated ECs. During the past two decades, several research groups have tried to elucidate a concise characterization of EPCs without good successes. The main reason resides in the surface markers used to phenotype EPCs which are common between haematopoietic stem cells and differentiated ECs.

In a first instance, scientists believed that EPCs arose from haemangioblasts like other haematopoietic cells [12] and this idea was supported by several* in vitro* and* in vivo* assays. Indeed embryonic stem cell- (ESC-) derived blast colony forming cells (BF-CSCs), considered an* in vitro* haemangioblast, were able, after VEGF-A or bone morphogenic protein-4 (BMP-4) stimulation, to differentiate into endothelial or haematopoietic cells, respectively [13]. Later other* in vivo* experiments in mice and zebrafish embryos [14, 15] sustained the role of haemangioblasts in EPCs development. On the contrary, few studies affirmed that endothelial and haematopoietic cells did not share a common precursor cell [16]. So the presence of haemangioblast is still unclear; moreover more studies support the idea of a common cell that could differentiate into endothelial or haematopoietic cells under appropriated stimuli. Progenitor cells are different from stem cells because they lack their self-renewal capability, but EPCs maintain many stem cells abilities, including self-renewal, clonogenicity, and differentiation. Furthermore EPCs express some undifferentiated and differentiated stem cells lineage markers such as CD133, fibroblast growth factor receptor (FGFR), CD146, CXCR4, ckit, vascular endothelial cadherin (VEcadherin), platelet endothelial cell adhesion molecule 1 (CD31), von Willebrand factor (vWF), angiopoietin 1 receptor precursor or tunica intima EC kinase (Tie2/TEK), and CD38 [17–19]. Unfortunately, these are surface markers that do not identify and discern exclusively EPCs.

According to the first studies, EPCs were identified as cells positive for both CD34 (HSC's marker) and VEGFR2 (endothelial marker). Several groups suggested that peripheral blood and BM-derived CD34+ cells are able to behave as ECs; indeed they can express endothelial specific markers and they show endothelial lineage abilities both* in vitro* and* in vivo* [20–22]. In contrast Case et al. [23] affirmed that CD34+/CD133+/VERGR2+ cells were not EPCs and that these cells, however, could acquire an endothelial phenotype. Furthermore the authors affirmed that CD34+/CD133+/VERGR2+ cells expressed also the CD45 marker, thus representing haematopoietic progenitors and not EPCs. Moreover Madeddu et al. [24] affirmed that the population CD34+/VEGFR2+ is enriched in EPCs, while Friedrich et al. [25] showed that CD133+/CD34/VEGFR2+ cells were able to regenerate vasculature structures and represented immature EPCs which differentiated in mature ECs. Taken together, these contradictory studies suggest that only a part of CD34+ cells can represent EPCs and, probably, EPCs derive from a subpopulation of CD34 positive cells. Since CD34 as well as VEGFR2 can be also expressed by mature ECs, other markers must be used to determinate and isolate EPCs. One of these is CD133. CD133 is also known as AC133 or prominin and it is a highly conserved orphan receptor that could represent another EPCs marker. Although a unique EPCs marker is still undetermined, it is commonly accepted that EPCs, residing in BM or immediately circulating, express CD133/CD34/VEGFR2 and are also known as circulating ECs or CECs, while circulating EPCs maintain CD34 and VEGFR2 but lose CD133 and acquire other endothelial lineage markers [10]. Indeed Peichev et al. [26] asserted that peripheral circulating CD34+/VEGFR2+ cells, that coexpress CD133, are immature EPCs. When they are recruited in the site of damage, these cells become mature under cytokines stimuli and they lose CD133 maintaining CD34 and VEGFR2. But CD133 is also expressed by haematopoietic stem cells like immature EPCs and it is missed on mature ECs [27].

These opposing data suggest that it is difficult to define and isolate exactly EPCs especially in the field of oncology where EPCs are often identified as circulating CD133+ cells. Moreover, the possibility to identify EPCs is strictly correlated with the technics used to evaluate their surface markers.

The number of circulating EPCs is low and variable according to the method and antigens used to identify these cells [28]. To solve this problem, most authors use different culture assays to increase the amount of EPCs from peripheral blood. All protocols for EPCs isolation differ concerning the culture time and most of them are short term protocols (47 days) [29]. Overall, we can count three methods for culturing EPCs* ex vivo*. In the first method, peripheral blood mononuclear cells (PBMCs) are seeded on dishes precoated with proteins that mimic extracellular matrix (ECM) such as fibronectin, collagen, or gelatin [30]. After short term culture of 47 days, a step essential to remove differentiated ECs and monocytes/macrophages, nonadherent cells are reseeded on dishes. These cells will form in one week colony forming units (CFU) composed of round cells in the centre with spindle shaped attaching cells and present ECs phenotypical characteristics and markers such as CD31, VEGFR2, VEcadherin, and TIE2.

Moreover, Hur et al. [31] demonstrated that the core of these colonies was formed also by CD3+, CD31+, and CXCR4+ cells, i.d. T cells defined as angiogenic T cells. The cells that form CFU take the name of CFU-ECs or early EPCs [30–32]. They have lowered proliferation ability, survive in culture condition for not less than 3040 days, and must express not only endothelial markers but also CD14 (myeloid marker) and CD45 (panleukocytic marker) [28]. With the second method, PBMCs are cultured in a medium supplemented with proangiogenic cytokines for 47 days and later nonadherent cells are eliminated.

The adherent cells are able to sustain angiogenesis* in vivo* and, for this reason, they are defined as circulating angiogenic cells (CACs). They show an endothelial phenotype binding specific endothelial antigens such as lectins as Ulex Europaeus Agglutinin 1 (UEA1) and Bandeiraea Simplicifolia (BS1) lectins, express CD31, Tie2/TEK, VEcadherin, and von Willebrand factor (vWF), and could take up acetylated low density lipoprotein/acLDL. Moreover CACs seem similar to CFUECs because they share marker profile and* in vitro* properties. For this reason, both cell populations have been often confused or not well discriminated and both CACs and CFUECs are defined as EPCs [28–30]. With the last method, we can identify endothelial colony forming cells (ECFCs); since they come from long term culture (until 2 to 3 weeks) of early EPCs, they are also called “late” or “outgrowing” EPCs. The late EPCs acquire mature ECs phenotype expressing markers like CD31, VEGFR2, VEcadherin, and vWF, with CD133 and CD34, and they have a cobblestone-like shape typical of mature ECs. Finally, outgrowth EPCs would differentiate into mature ECs to begin and promote angiogenesis and vasculogenesis [28–30]. To elucidate the complexity of several types of EPCs, Prater et al. [33] suggested an interesting hypothesis: CACs were the largest part of cultured EPCs; further they supposed that ECFCs took part in the circulating ECs population and that CD45+ haematopoietic progenitor cells cross with CFUECs in an undetermined manner.

Recent studies demonstrated that ECFCs do not express hematopoietic markers, showing that they do not have a hematopoietic origin. Piaggio et al. [34] proved that ECFCs, unlike CFU-ECs, lack the expression of CD45 and CD14 markers and they also demonstrated that CFU-ECs presented the same mutations of circulating leukocytes from patients with myeloproliferative disease, whereas ECFCs did not. This result highlighted that ECFCs have a different origin from hematopoietic cells. Raemer et al. [35] demonstrated that these ECFCs are able to costimulate T cells as well as monocytes better than mature ECs, such as HUVEC. Finally, Prokopi et al. [36] showed that the expression of mature ECs antigens such as CD31, VEGFR2, and Von Willebrand factor by CFU-ECs is mainly due to a platelet contamination within buffy coat at the moment of cells isolation. Proteomic studies proved that early EPCs uptook endothelial markers from platelets, not because they expressed these endothelial markers themselves. Thus, all these recent studies underlined that only few EPCs are able to form vascular network* in vivo* and show a higher proliferative ability and these cells are represented by ECFCs [37]. So, ECFCs, which represent only 1% of all the circulating EPCs, are supposed to be true EPCs and they are better described as angiogenic macrophages [38].

## 3. Key Pathways in Vasculogenesis

There are several pathways which regulate haemangioblasts differentiation and vasculogenesis during embryo development but also EPCs recruitment in postnatal life. These pathways may also be altered in some pathological conditions such as malignancies.

### 3.1. VEGF and Other Cytokines

Vascular endothelial growth factor plays a key role in regulating both angiogenesis and vasculogenesis.

There are five different isoforms of VEGF generated as a result of alternative splicing from a single VEGF gene (VEGFA, -B, -C, -D, and placenta VEGF also known as PIGF) which interacts with three main tyrosine kinase receptors (VEGFR1, -2, and -3) [39]. The activation of VEGFR induces the mobilization of EPCs towards injured sites, and here the VEGF pathway stimulates their differentiation into mature ECs.

In particular VEGFA and VEGFR1, also known as Flt1, and VEGFR2, also known as Flk1, are expressed during embryo life [40].

VEGFA and its receptors, VEGFR1 and VEGFR2, are presented early in embryonic development. VEGFA is expressed in the extraembryonic endoderm and mesoderm during the formation of blood islands and it is also secreted within the intraembryonic endoderm. VEGFR2 is an early marker of endothelial and hematopoietic precursor cells in blood islands. Shalaby et al. [41] demonstrate that the expression of VEGF and VEGFR is necessary for a correct vasculogenesis process: the lack of VEGFR2 induces the embryo death due to the failure of vasculogenesis and hematopoiesis. Knockdown of VEGF signaling during embryo development implied a decreased cell migration and the consequent defective formation of blood islands. Similarly, deletion of VEGF and VEGFR1 results in embryo lethality due to problems in vascular development. In particular, the lack of a single VEGF allele results in embryo death caused by an improper development of the dorsal aorta and an altered haematopoiesis; the deletion of VEGFR1 induces an inappropriate localization of haemangioblasts in the blood islands; instead the knockdown of VEGFR3 appears to be mainly involved in lymphatic vessels development [42].

Platelet-derived growth factor (PDGF) is strongly associated with VEGF pathway, and it is involved in cell migration during vascular development.* In vivo* and* in vitro* studies showed that activating mutations of PDGF receptor (PDGFR) in embryo promote vascular development; instead PDGF antagonists inhibit vasculogenesis and tumor growth in rat model [43].

Another important cytokine involved in vascular development is Transforming Growth Factor *β* (TGF*β*).

Many studies reported its critical role showing how the lack TGF*β* results in disorders during yolk sac vasculogenesis and the death of half of the mutant embryos at week 9.510.5; instead the remaining embryos die weeks later due to inflammation [44, 45]. Also the knockdown of TGF*β* receptor causes embryonic death at week 10. Stromal cell-derived factor 1 (SDF1) is an important chemokine in EPCs and HSCs mobilization, not only in wound healing and tissue damage but also during organogenesis and embryo vascular development [46].

Endoglin is a transmembrane receptor for TGF*β*, which coexists with TGF*β* receptors and which is highly expressed in endothelial cells. Mice lacking endoglin die at week 11.5 for defects to vascular system and atypical cardiac development [47]. Liu et al. [48], using mice knocked out for endoglin, demonstrated that endoglin is necessary to ensure the organization of haemangioblasts into tubular structures and that endoglin is required for a correct vasculogenesis process depending on VEGF.

These cytokines have also a crucial role in regulating tumor vasculogenesis contributing to the angiogenic switch in both solid and haematological malignances. The expression of these molecules changes from different stages of tumor growth, remission, and relapse. Many studies reported the altered secretion of proangiogenic molecules such as VEGF, FGF, PDGF, EGF, TGF*β*, and angiopoietin within the tumor microenvironment which contribute to EPCs mobilization and differentiation and ECs proliferation. So, in tumor patients a great percentage of ECs undergo mitosis versus the very lower 0,01% of healthy adults, highlighting the idea of an enhanced vessel growth in these pathological conditions [49].

### 3.2. Notch

Notch signaling is involved in many developmental phases but also in neoplastic processes such as tumor angiogenesis and vasculogenesis, Endothelial to Mesenchymal Transition (EMT), drug resistance, and metastasis. There are four different Notch transmembrane receptors (Notch1, -2, -3, and -4) and five ligands belonging to two different families, Jagged1 and Jagged2, and the ones belonging to the Delta family Delta-like 1 (DLL1), DLL3, and DLL4. The bond of a Notch ligand to the receptor induces three proteolytic cleavages with the formation of the so-called Notch transcriptional domain which translocate into the nuclei and together with other transcriptional factors forms the “Notch transcriptional complex” (NTC). Thus Notch acts as a transcriptional regulator, inducing the expression of many genes related to differentiation and survival, including hairy/enhancer of split (HES), the family of helix-loop-helix transcription factors, cyclin D, and cMyc [50]. Many studies reported that the lacking of Notch receptors or their ligands is involved in vascular defects during embryo development and also in ECs maturation and migration. Fischer et al. demonstrated that the Notch target genes Hey1 and Hey2 are required for a correct embryo vascular development. The knockdown of Hey1 alone induces cardiac defects and a high probability of postnatal death; instead the loss of Hey2 does not result in any particular defects; but the loss of both Hey1 and Hey2 causes embryonic lethality at week 9.5 due to a defective vascular remodeling and the presence of truncated vessels responsible for abundant hemorrhages. This study highlights the importance of these two target genes as principal effectors of vasculogenesis regulated by the Notch pathway [51]. Also the deletion of Notch receptors 1 and 4 and Notch ligand DLL4 results in embryonic death and alteration of vasculature development [52]. Many authors also demonstrated the important role of Notch signaling in the regulation of tumor vasculogenesis and angiogenesis. Alteration of the expression of Notch ligands Jagged1, which enhances tumor angiogenesis, and DLL4, which acts as negative regulator of tumor vasculogenesis, has been discovered in various tumors [53].

### 3.3. Hedgehog

Hedgehog pathway is involved in embryo development and vasculogenesis and regulates cell proliferation and migration. Hedgehog family includes three different ligands: sonic hedgehog (Shh), Indian hedgehog (Ihh), and desert hedgehog (Dhh). The bind of hedgehog ligands to their transmembrane receptor, Patched, induces a conformational change that removes the repression of the transmembrane Smoothened protein (Smo). The activation of Smoothened regulates the expression of GLI transcriptional activators which activate Hedgehog target genes themselves [54]. All ligands are involved in vasculogenesis. Ihh ligand seems to be necessary for an appropriate blood island formation in early stage of yolk sac vessel development and of lumen formation in murine embryo; mice knocked down for Ihh showed defective vasculogenesis during yolk sac development resulting in embryos death [55]. Dhh ligand instead is activated during embryo gastrulation and is expressed in mature yolk sac mesoderm. Dhh role seems to be similar to those of Ihh and might compensate Ihh inactivation. Shh regulates angiogenesis in different tissue in late phases of embryogenesis. FGF activates hedgehog pathway through Shh and stimulates the expression of VEGF and Ang2. Many studies reported that Shh is involved in regulating EPCs recruitment from bone marrow and in their differentiation into mature endothelial cells [56]. Finally, also an inactivation of Smo protein, which is the main downstream effector of hedgehog pathway, is necessary for a functional vasculogenesis and Vokes et al. [55] demonstrated that mice knocked down for Smo die at week 7.58.0 during embryo life. Maybe hedgehog signaling is able to regulate vasculogenesis through the activation of its target Bone Morphogenetic protein 4 (Bmp4). Inactivation of Bmp4 induces defective vascular development. Recently, a strict relationship between Hh pathway and malignancies has been proved. Chen et al. [57] demonstrated that tumor cells express hedgehog ligands and this signaling pathway is active in the tumor microenvironment. In particular, the activation of canonical hedgehog pathway induces fibroblasts to secrete VEGF and other proangiogenic growth factors, which enhances not only tumor angiogenesis but also the mobilization of EPCs from BM niches.

## 4. Homing and Mobilization of ECPs

In physiological conditions, the peripheral blood number of circulating EPCs is low because they are located in stem cell niche in the BM where the microenvironment governs EPCs mobilization through high level of stromal cell-derived factor1 (SDF1) and hypoxic conditions. During trauma and wound healing or during cancer progression when the increase of tumor mass causes the angiogenic switch with the production of proangiogenic cytokines and factors (VEGF, basic FGF, PDGF, erythropoietin or EPO, granulocyte colony stimulating factor, or GCSF), BMEPCs, responding to stimuli, are mobilized from BM and they acquire typical characteristics of CECs and arrive at injury site and here they differentiate into mature ECs. The activation of different molecular pathways occurs in EPCs, VEGF pathway being the most important. VEGF is able to activate endothelial nitric oxide synthase (eNOS) that produces nitric oxide [58]. It regulates the activation of matrix metalloproteinases (MMPs), in particular MMP9.

Activated MMP9 promotes the cleavage of membrane bound stem cell cytokine mKitL by stromal cells in the BM niche; thus soluble kit ligand sKitL is released. sKitL stimulates cKit+ EPCs inducing cell mobilization from BM niche into peripheral blood. This migration elicits EPCs activation acting in a transition from a quiescent to a proliferative stage [59]. Moreover VEGF regulates SDF1 and its receptor (CXCR4) that are discovered to be chemotactic for EPCs promoting the recruitment of EPCs into the vascular zone. SDF1 needs other signals to recruit EPCs, such as VEGF. Furthermore SDF1 helps EPCs to remain within the vasculature site [60]. Overall, VEGF interacts with MMP9 and SDF1 developing EPCs release into peripheral bloodstream; consequently in cancer patients, the increase of VEGF levels leads EPCs mobilization from the BMniche to the bloodstream [61, 62]. Moreover, integrins regulate the retention of EPCs in the BMniche, particularly *α*_4_ and *β*_3_. Indeed studies* in vitro* and* in vivo* show that *α*_4_ and *β*_3_ alterations favor the migration of EPCs from the BM microenvironment to the peripheral blood [4]. In addition, deletion of *α*_4_ integrin specifically inhibits EPCs and haematopoietic stem cells mobilization to BM but not to spleen, showing that different integrins expressed on EPCs promote their homing to different tissues [63]. Other studies [64] demonstrate the implication of other integrins in the neovascularization such as *β*_2_ integrins and *β*_1_ integrins. *β*_2_ integrins (also known as CD11/CD18) are found on haematopoietic stem cells and on peripheral-derived EPCs and are involved in cell-cell adhesion and EPCs transmigration through the blood vessels; in fact different studies support that *β*_2_ integrins activation promotes the neovascularization ability of EPCs* in vivo*. But the inhibition of these integrins blocks partially EPCs homing, underlining that there are other integrins involved in EPCs mobilization to damage tissue or tumor mass [64, 65]. *β*_1_ integrins are expressed on both ECs and haematopoietic cells. This family governs EPCs mobilization to angiogenic sites and interacts with activated ECs through vascular cell adhesion protein (VCAM) and cellular fibronectin. In particular, *α*_5_ *β*_1_ is overexpressed on circulating EPCs to promote the bind with vessels fibronectin, while *α*_6_ *β*_1_ is also implicated in endothelial tubes formation during neovasculogenesis [66]. Moreover, Qin et al. [67] reported that several integrins are expressed only in circulating and not differentiated EPCs, while others are expressed during EPCs differentiation. For example, during their differentiation, EPCs express *α*_4_, *α*_5_, and *α*_*v*_ integrins that interact with fibronectin underlining the important role of fibronectin in EPCs maturation.

Going into details, once EPCs leave their niche, they are activated and interact with ECs by integrins to ensure their transmigration through endothelial monolayer. But to invade blood vessels, EPCs produce and secrete MMP9 that promotes ECM disruption and its remodelling. Once EPCs arrive at the site of damage or near tumor mass, they can take part in neovascularization in three ways: (1) EPCs are directly incorporated in new vessels, (2) EPCs differentiate in mature ECs, and (3) EPCs produce and secrete proangiogenic factor and cytokines with paracrine effects such as VEGF, bFGF, SDF1, PDGF, insulin-like growth factor (IGF1), macrophage inflammatory protein 1a, and monocyte chemotactic protein 1 (MCP1) [68]. But other molecules stimulate EPCs homing such as granulocyte/macrophage colony stimulating factor (GMCSF) or EPO. Indeed Takahashi et al. [69] demonstrated that animals treated with exogenous GMCSF showed an increase of circulating EPCs; similarly, Bahlmann et al. [70] proved* in vitro* and in human peripheral blood that recombinant human erythropoietin (rhEPO) is able, in a dose dependent manner, to enlarge functionally active EPCs.

In conclusion, EPCs, which leave the BMniche and are released into the bloodstream, beyond the BM microenvironment control, begin their differentiation, under different stimuli, into CECs to finally become mature ECs activating important pathways involved in injuries repair and tumor progression.

## 5. EPCs in Haematological Malignancies

CECs are associated with neovasculogenesis in various malignant disorders. Several studies have shown that vasculogenesis in haematologic tumors is closely related to the existence of an adult haemangioblast population that leads to the neoformation of blood vessels in these tumors [71].

As demonstrated by Folkman's group in 1997 there is an increase in BMMVD in acute lymphoblastic leukemia patients suggesting a correlation between angiogenesis and leukemia progression [72]. MVD increase and high levels of numerous proangiogenic factors have been reported in acute and chronic leukemia and occasionally were correlated with worse survival [73]. The same findings on the role of vasculogenesis were demonstrated later in myeloma, non-Hodgkin lymphoma (NHL), and myelodysplastic syndromes (MDS).

### 5.1. Acute Leukemia

Acute myeloid leukemia (AML) is a heterogeneous haematological disease characterized by the clonal expansion of myeloid blasts in the peripheral blood and BM. AML is the common form of acute leukemia in adults.

The vessels recruitment has a role in the pathogenesis of AML. Indeed Hussong et al. conducted a study on AML patients that showed that MVD is greater in leukemic BM biopsy samples from untreated patients compared to those of normal controls [74]. This study confirmed that a correlation exists between increased BM vascularity and overall survival of leukemia patients and that a higher MVD can contribute to refractory disease [75]. Furthermore, Fiedler et al. [76], investigating the vascular niche, found increased expression of VEGF, as well as VEGFR1 and VEGFR2, in leukemic blasts from patients with AML.

Cultured endothelial human umbilical cord cells (HUVECs) under VEGF stimuli produce a dose dependent increase in GMCSF, suggesting paracrine mechanisms [77]. ECs support adhesion and transmigration subsets of CD34+ normal HSPCs. Several* in vitro* studies with HUVECs in cocultures with promyelocytic leukemia cells (HL60) exposed to cytarabine showed that these ECs have a protective effect on leukemic myeloblasts. However, recent studies suggest that leukemia cells may have reciprocal effects of enhancing the proliferation of ECs establishing a cyclic positive feedback loop which favors the potential for refractory and relapsed disease [78].

Finally, an increase of circulating angiogenic factors such as VEGF and angiopoietin (Ang2) and the increased angiogenesis in the BM are indicators of high risk of recurrence of disease and early mortality as shown by Madlambayan et al. [79] in mononuclear cells of the BM in AML patients newly diagnosed before treatment. However, the meaning of Ang2 in AML is complex and most likely influenced by VEGF [72, 79].

Acute lymphocytic leukemia (ALL) is the most common form of leukemia in children and is caused by the accumulation of lymphoblasts in the BM. In adult ALL, some studies show the role of angiogenesis and vasculogenesis with increasing evidence that angiogenesis markers play a role in the pathogenesis of patients with ALL.

Hou et al. [80] found a correlation between high levels of cellular VEGF at diagnosis of ALL and the number of leukemic blasts as well as the behavior of the surrounding microenvironment. Conversely at the time of remission the BM angiogenic switch takes place with the secretion of VEGF and other proangiogenic factors in the serum [81]. In fact it can be difficult to distinguish the production and secretion of angiogenic factors reactive compared to a true reflection of the pathophysiological process [82].

Perez-Atayde et al. [72] demonstrated a significant increase of MVD in the BM of 40 children with ALL compared to that in control marrows.

The three-dimensional structure of normal marrows which show single, straight, nonbranching vessels, and leukemic marrows with complex, arborizing branching vessels has been studied. The average number of blood vessels in the BM is significantly higher than in those, normal of the same age [83].

Similarly, serum levels of bFGF but not VEGF were significantly elevated in these children. However, other studies reported increasing levels of both VEGF and bFGF in patients in remission or they failed to detect significant differences in MVD at presentation or remission from children in poor prognostic groups of ALL [84].

### 5.2. Chronic Leukemia

Chronic lymphocytic leukemia (CLL) is blood and BM disease that usually gets worse slowly. CLL is one of the most common types of leukemia in adults.

In this malignancy, experimental and clinical data suggest that angiogenesis may have a role in the pathogenesis and progression of the disease.

Aguayo et al. [83] showed an increase of the MVD in the BM of patients with CLL, which can reach up to three times compared to healthy controls. A study performed in 2010 of 170 patients with CLL explained that CECs and EPCs are more numerous in CLL than in healthy controls and that this increase correlated with a more aggressive disease. In particular they demonstrated that gene expression profiling of separated waste CECs expressed a molecular model of probable derivation from CLLs; these cells were characterized by an increased proliferation and proangiogenic function but also decreased adhesion to the ECM and the consequent increase in survival compared to normal subjects [85].

Ultimately CECs may be somewhat related to the angiogenic switch which characterizes the transition to a more aggressive stage of the disease; so CECs could represent a new prognostic indicator. Further studies will be used to clarify whether, in CLL patients, CECs can be used to evaluate the effectiveness of treatment and whether patients with elevated CECs may benefit from treatment with the new antiangiogenic drugs [85].

Chronic Myelogenous Leukemia (CML) is a clonal disorder of a pluripotent stem cell. The disease accounts for almost 15% of leukemia cases and may occur at any age.

Lundberg et al. [86] demonstrated that the chronic myeloproliferative diseases, chronic myeloid leukemia, and myelofibrosis were associated with increased MVD in the BM compared to healthy subjects. Data also suggested that VEGF, increased in CML, could be an important signaling molecule to recruit EPCs in these conditions. Also the architecture of the vascular environment in myeloproliferative disorders is much more tortuous and branched than normal. Moreover, the number of vessels and their ramifications increases proportionally with worse prognosis [86].

### 5.3. Non-Hodgkin's Lymphomas

These represent a large group of lymphoid tumours, most commonly of B-cell origin, whose clinical presentation and natural history are more variable. The neovessel formation is different depending on the type of lymphoma: it is higher in aggressive subtypes such as in the involved lymph nodes of patients with BCLL (small lymphocytic lymphoma) and Burkitt lymphomas; it is lower in diffuse large B-cell lymphomas and in indolent follicular lymphoma [87]. Preclinical studies have already shown the importance of detecting the level of circulating EPCs during the various stages of malignancies [8].

Igreja and collaborators sought to define the relative importance of circulating EPCs versus EPCs located in the affected tissues. This study performed on CD133+/CD34+/VEGFR2+ EPCs in PB and lymph nodes of patients with NHL highlighted an increased number of circulating EPCs in NHL younger patients with more aggressive disease and reduced levels of EPCs in patients with incomplete response to treatments. Lymph nodes EPCs (LNEPCs) were not only found around the vessels but also could be found scattered throughout the tissue. Genes expressed by LNEPCs encode proangiogenic factors and chemoattractant cytokines that contribute to the expansion of the lymphoma mass. The concomitant presence in NHL patients of both circulating EPCs and LNEPCs indicates that the development of the disease can be monitored by new surrogate markers probably involved in the pathogenesis and vasculogenesis of lymphomas [88].

### 5.4. Myelodysplastic Syndromes

Myelodysplastic syndromes (MDS) are a heterogeneous group of bone marrow failure disorders characterized by an inexorable but variably gradual trend of progressive cytopenias and transformation to AML in approximately 30% of cases [89]. The formation of new vessels is a fundamental process for the development of MDS [90]. Della Porta et al. [91] showed that levels of CECs (CD45 gate as D146+/CD34+ cells) increased in MDS patients compared to CECs controls and that this was particularly true for those without excess blasts. The distinction between EPCs and mature ECs was made by identifying the expression of CD133 in the progenitor to mature ratio. This was higher in patients at diagnosis compared to individuals at followup, suggesting that, in MDS, the mobilization of ECs in the BM is greater in the early stages and then decreases during the disease progression and indicating that the majority of CECs in MDS, but not in healthy individuals, consist of precursor cells. So medullary ECs play a key role in tumor transformation [92]. Furthermore BM microvessel density was found to be related to the CECs and higher in low risk patients. The study by Della Porta et al. [91] adds more guidance to the use of therapies designed to restore a normal interaction between haematopoietic progenitor cells and BM microenvironment.

### 5.5. Multiple Myeloma

Multiple myeloma (MM) is a debilitating malignancy characterized by the clonal proliferation of neoplastic plasma cells in the BM and a subsequent excess of monoclonal paraprotein in blood and/or urine [9]. In the BM of MM patients HSCs are recruited and induced to differentiate into ECs by the angiogenic cytokines such as VEGF, bFGF, and IGF present in the microenvironment and they participate in the formation of new vessels [93]. Several studies have investigated the role of CECs and EPCs in MM progression. Vacca et al. [94] in 1994 had already shown the increases in parallel with the MVD and the risk of active disease in patients with MM. A study of 75 patients with multiple myeloma showed higher levels of circulating EPCs (defined as D45/CD34+/CD133+/CD31+ cells) in patients with active disease increasing, respectively, from Durie and Salmon phase I to phase III compared to healthy controls.

Circulating EPCs were instead reduced after therapy in patients who achieved at least a partial response [95]. Zhang et al. [96] studying EPCs and CEC by RTPCR and flow cytometry, respectively, confirmed the higher levels of the two cell types in MM patients compared to healthy controls. In particular CECs were six times higher in patients than in controls proportionally to the increase of two markers of disease activity such as serum proteins M and *β*_2_ microglobulin. Also a study by the Mayo Clinic showed a strong association between angiogenesis grade and density. In fact, patients with ≤50 microvessels/field survived 5.1 years, while those with more than 50 survived only 2.6 years (*p* = 0.004) and concluded that bone marrow angiogenesis can be a predictor of poor survival in newly diagnosed myeloma [97].

### 5.6. Conclusions and Therapeutic Perspectives

The use of EPC has been proposed as a therapeutic target in different diseases, for example, the role of EPC in the treatment of cardiovascular diseases. A recent study investigated the possibility of autologous EPCs therapy for therapeutic revascularization and vascular repair in ischemic pathologies [98].

Moreover, during cancer progression, vasculogenesis-mediated EPC would represent an interesting therapeutic target and could act as a potential reference point for the synthesis of new and more effective antiangiogenic drugs [99].

However these therapies require a full knowledge of the mechanisms underlying the recruitment of EPCs [98].

The numerous discoveries of MVD in different tumor diseases have opened a way to EPC therapies, but despite being effective, the antivessel treatment in many patients fails to halt tumor progression.

BM-derived EPC-mediated vasculogenesis is a very important event in promoting the “angiogenic switch.” In haematological malignancies many investigations are still ongoing to characterize more precisely the finest EPCs associated with cancer [10, 99–101].

These studies will inform new perspectives for the rational use of antivascular therapies in blood cancers.
